# Notch Signaling during Oogenesis in *Drosophila melanogaster*

**DOI:** 10.1155/2012/648207

**Published:** 2012-05-03

**Authors:** Jingxia Xu, Thomas Gridley

**Affiliations:** ^1^The Jackson Laboratory, Bar Harbor, ME 04609, USA; ^2^Department of Molecular and Biomedical Sciences, University of Maine, Orono, ME 04469, USA; ^3^Center for Molecular Medicine, Maine Medical Center Research Institute, Scarborough, ME 04074, USA

## Abstract

The Notch signaling pathway is an evolutionarily conserved intercellular signaling mechanism that is required for embryonic development, cell fate specification, and stem cell maintenance. Discovered and studied initially in *Drosophila melanogaster*, the Notch pathway is conserved and functionally active throughout the animal kingdom. In this paper, we summarize the biochemical mechanisms of Notch signaling and describe its role in regulating one particular developmental pathway, oogenesis in *Drosophila*.

## 1. Introduction

Utilized by the simplest metazoans through mammals, Notch signaling is an evolutionarily conserved signaling pathway that is required for embryonic development, cell fate specification, and stem cell maintenance [1–5]. Notch signaling selects among preexisting cellular potentials to specify different cell fates and activate different programs through either promoting or suppressing differentiation, proliferation, survival, and apoptosis [6, 7]. In humans, mutations in this pathway cause inherited genetic diseases such as Alagille syndrome, spondylocostal dysostosis, Hadju-Cheney syndrome, Tetralogy of Fallot, familial aortic valve disease, and cerebral autosomal dominant arteriopathy with subcortical infarcts and leukoencephalopathy. Dysregulation of Notch activity also is associated with T-cell acute lymphatic leukemia and other cancers (e.g., pancreatic, ovarian, colon, and brain tumors) [3, 8–13].

## 2. Notch Receptors and Ligands

The *Notch* gene was discovered by Morgan and colleagues, who observed that X-linked dominant mutations in *Drosophila* caused irregular notches at the wing margin [14, 15]. Later, Poulson found that the absence of *Notch* activity in the embryo resulted in the overproduction of neural tissue at the expense of epidermal tissue [16]. This phenotype was termed *neurogenic* and was later shown to be a characteristic phenotype of several other *Drosophila* mutants. This defined the *Drosophila* Notch pathway as a cascade of neurogenic genes that control the formation of the fly nervous system [17]. However, *Notch* mutants also exhibit several other defects in embryonic and adult tissues, which indicates that this pathway is involved not only in the development of the nervous system but also in cell fate decisions. Today, with subsequent identification of orthologs for *Notch* in *Caenorhabditis elegans* and higher vertebrates [18–20], it has been shown that the Notch pathway regulates cell fate decisions, affecting almost all cells of complex animal tissues for proper final differentiation.

One Notch receptor gene exists in *Drosophila*, two in *C. elegans* (*Lin-12* and *Glp-1*) and four in mammals (*Notch1*, *Notch2*, *Notch3,* and *Notch4*). The *Notch* gene encodes a 300 kDa single-pass (Type 1) transmembrane receptor. In mammals, the Notch receptors are expressed as propeptides that are constitutively cleaved in the trans-Golgi network by furinlike proteases at Site 1 (S1) [21, 22]. Cleavage results in the extracellular/lumenal N-terminal fragment and the transmembrane domain/intracellular domain/C-terminal fragment. A heterodimer is formed through a noncovalent Ca^++^-dependent interaction between these two domains and is targeted to the plasma membrane to form the receptor.

The Notch receptors have several conserved domains [21]. The extracellular domain has 29–36 tandem epidermal-growth-factor- (EGF-) like repeats, some of which are required for ligand interaction [23]. For example, repeats 11–12 mediate productive interactions with ligand presented by neighboring cells (*trans*-interactions), while repeats 24–29 mediate inhibitory interactions with ligand coexpressed in the same cell (*cis*-interactions) [24]. Many of the EGF repeats bind calcium, which determines the structure and affinity of Notch receptors for their ligands [25]. Following the EGF repeats is a unique negative regulatory region (NRR), which is composed of three cysteine-rich Lin-12/Notch repeats (LNRs) and a heterodimerization domain (HD). The NRR is conserved in all Notch receptors and prevents receptor activation in the absence of ligand. The single transmembrane domain has a stop-translocation signal that contains three to four Arg/Lys residues. The Notch intracellular domain (NICD) is comprised of the RAM (RBPJ association molecule/module) domain, which consists of 12–20 amino acids centered around a conserved Trp-Xaa-Pro (WxP) motif [26]. This motif has a high binding affinity to CSL (an acronym for CBF/RBPJ in vertebrates, *Suppressor of Hairless* in *Drosophila*, and *Lag-1* in *C. elegans*) [27]. A linker containing a nuclear localization sequence (NLS) connects the RAM domain to seven ankyrin repeats (ANK domain). The ankyrin repeats are involved in protein interaction with CSL and facilitate interaction with other proteins such as deltex homologs and NUMB, which are important cytosolic regulators of the Notch pathway. Both the RAM and ANK regions of NICD are important for CSL-mediated Notch signaling. Following the ANK domain is another NLS and an evolutionarily divergent transactivation domain (TAD). The C-terminus of the Notch receptors is comprised of conserved Pro/Glu/Ser/Thr-rich motifs (PEST). These motifs regulate protein turnover of the NICD [28, 29]. *Drosophila* Notch also contains a glutamine-rich OPA repeat, which is composed of repeating units of the sequence triplet CAX where X is either G, A, or T [21, 30].

Based on their domain composition, the ligands and potential ligands of Notch receptors can be divided into different groups [21]. The canonical DSL (Delta and Serrate from *Drosophila* and Lag-2 from *C. elegans*) ligands conduct the majority of Notch signaling effects. Like Notch receptors, the DSL ligands are also type 1 transmembrane proteins, although they have much smaller and less conserved intracellular domains than the Notch receptors. The classical Notch ligands all share a similar structure [21]: an N-terminal DSL domain, specialized tandem EGF repeats called the DOS domain (Delta and OSM-11-like proteins [31]), EGF-like repeats, a transmembrane segment, and a short (approximately 100–150 amino acids) cytoplasmic domain [32]. The ligands can be divided into two families: homologs of *Drosophila* Delta protein (DLLs, Delta-like 1, 3, and 4 in mammals) and homologs of *Drosophila* Serrate (JAGs, Jagged 1 and 2 in mammals) [6, 7]. This division is based on the presence or absence of a cysteine-rich domain. Specifically, the JAG ligands have the cysteine-rich region proximal to the transmembrane segment. Compared to the Delta-like ligands, JAG1 and JAG2 have almost twice the number of EGF repeats, and some of these repeats contain conserved insertions of unknown function [33]. Within the same ligand type, the intracellular region of the Notch ligand is well conserved through evolution. However, different ligand types have distinct cytoplasmic domains.

The DSL domain is characterized by the conserved specific spacing of six cysteines and three glycines. Both the DSL and DOS domains are involved in receptor binding, with the DSL domain involved in both *trans-* and *cis*-interactions with Notch receptors [7, 34–36]. Compared with the activating *trans*-interactions, *cis*-interactions between DSL ligands and Notch receptors inhibit Notch signaling [37–40] and play an important role in a subset of Notch-dependent developmental events [38, 39, 41].

The intracellular domain of JAG1, as well as DLL1 and DLL4, contains a PDZ-ligand domain, which is required for interactions with PDZ-containing, membrane-associated proteins that play a role in the organization of cell-cell junctions. PDZ stands for the three proteins first discovered to share the domain: postsynaptic density protein (PSD-95), *Drosophila* discs large tumor suppressor (DLG1), and zonula occludens-1 protein (ZO-1). Interaction with the PDZ domain is independent of interaction with the Notch receptor. For example, DLL-1/4 can recruit DLG1 at cell-cell junctions, which results in tightening cell contacts and a reduction in cell motility [42].

For noncanonical ligands, *C. elegans* and mammalian DSL-only ligands (lacking DOS, including diffusible ligands) may act alone or in combination with DOS coligands [31, 43]. Noncanonical ligands lack both DSL and DOS domains, such as the neural adhesion molecule CNTN1 (contactin1 or F3/contactin) [44], the related NB3 protein [45], and the EGF repeat protein DNER (delta/notch-like EGF-related receptor) [46], which may facilitate the activation of Notch receptors by DSL ligands and/or DOS coligands. The physiological function for these proteins in the Notch pathway is yet to be established.

## 3. Mechanisms of Canonical Notch Signaling

**Figure 1:**
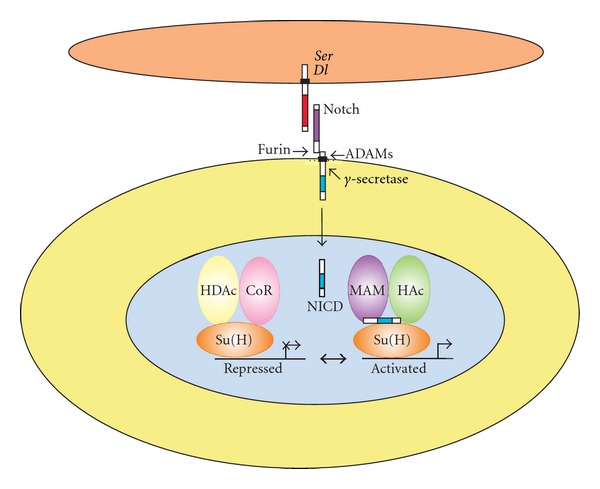
Core components of the canonical Notch signaling pathway in *Drosophila*. The two Notch ligands encoded by the *Serrate* (*Ser*) and *Delta* (*Dl*) genes (upper cell) interact with an adjacent cell expressing the Notch receptor. The Notch receptor is proteolytically cleaved by a Furin protease in the Golgi and exists at the cell surface as a proteolytically cleaved heterodimer consisting of a large ectodomain and a membrane-tethered intracellular domain. The receptor/ligand interaction induces additional proteolytic cleavages by ADAM-family metalloproteases and the gamma-secretase complex in the membrane-tethered intracellular domain. The final cleavage, catalyzed by gamma-secretase, frees the Notch intracellular domain (NICD) from the cell membrane. NICD translocates to the nucleus, where it forms a complex with the Supressor of Hairless (Su(H)) protein, displacing a histone deacetylase (HDAc)/corepressor (CoR) complex from the Su(H) protein. Components of an activation complex such as the Mastermind (MAM) protein and histone acetyltransferases (HAc) are recruited to the NICD/Su(H) complex, leading to the transcriptional activation of Notch target genes.

The core mechanism of canonical Notch signaling is the release of NICD as a transcriptional regulator from the membrane (Figure 1). This process is activated by ligand-receptor interactions, and is controlled at many different levels (reviewed in [6, 21]). Activation of the canonical Notch signaling pathway is mediated by regulated sequential proteolysis. In mammals, the Notch protein is glycosylated by POFUT1 (protein O-fucosyltransferase 1) to produce a functional receptor. After proteolytic cleavage by PC5/6/FURIN (paired basic amino acid cleaving enzyme) at site S1, Notch receptors are targeted to the cell surface as a heterodimer. The O-fucose is extended by the glycosyltransferase activity of FRINGE proteins (O-fucosylpeptide 3-beta-N-acetylglucosaminyltransferase, including lunatic, manic, and radical fringe in mammals), which regulate the ability of specific ligands to activate Notch receptors. The interaction with ligands leads to cleavage of Notch receptors by ADAM (a disintegrin and metallopeptidase domain) metalloproteases (ADAM10/Kuzbanian and ADAM17/TACE) at site S2, which is located about twelve amino acids before the transmembrane domain. In the absence of ligand, the S2 cleavage site is in a *β*-strand, deeply buried within the NRR [47]. After ligand binding, the Notch ectodomain is transendocytosed by ligand-presenting/signal-sending cells while the NICD is localized in signal-receiving cells [48]. Transendocytosis generates sufficient force to promote a conformational change that exposes S2 site for cleavage, which results in the generation of membrane bound intracellular Notch peptides (NEXT, for Notch extracellular truncation) [49, 50].

NEXT is a substrate for cleavage by the *γ*-secretase complex, composed of presenilin 1 and 2 as well as nicastrin and alphaprotein 1 [51]. *γ*-secretase cleaves NEXT progressively, starting at the S3 site near the inner leaflet and ending at the S4 site near the middle of the transmembrane domain. *γ*-secretase cleavage can occur at the cell surface or in endosomal compartments; however, cleavage at the membrane results in the more stable form of NICD. This processing event releases NICD, which constitutively translocates into the nucleus, where it interacts via its RAM domain with the primary nuclear effecter of Notch signaling, the DNA-binding protein CSL.

The mammalian homologue of CSL is called C promoter binding factor 1 (CBF1) or recombination signal binding protein for immunoglobulin kappa J region (RBPJ), which mediates canonical Notch signaling [52]. The constitutively expressed RBPJ binds to a specific sequence on promoters of Notch target genes and regulates their expression. In the absence of NICD, RBPJ associates with ubiquitous corepressor (Co-R) proteins and histone deacetylases (HDACs), thereby repressing the transcription of specific target genes. Molecular and phenotypic experiments have shown that CBF1 can interact with various corepressors, including NCoR/SMRT (nuclear receptor corepressor 2), MINT/SHARP/SPEN (SPEN homolog, transcriptional regulator), CIR1 (corepressor interacting with RBPJ-1), and Groucho/transducin-like enhancer complexes [21, 53]. Different RBPJ-associated repressor complex components assemble on different Notch target promoters, with variations in the arrangement of RBPJ binding sites and transcriptional repressor complex types, resulting in regulation of gene expression (reviewed in [54]).

Upon ligand-induced Notch activation, the released NICD translocates into the nucleus and binds to RBPJ, which is crucial for the switch from a repressed to an activated state. NICD first displaces corepressors from RBPJ to derepress promoters containing RBPJ binding sites. Subsequently associating with RBPJ, the ANK domain of NICD recruits the transcriptional coactivator mastermind like proteins (Maml1-3) to form an RBPJ/NICD/MAML ternary complex. Conformational changes among the RBPJ, NICD, and MAML proteins drive the folding of unstructured protein segments and facilitate binding of other specific coactivators to form an activator complex. General transcription factors are recruited, such as the histone acetyltransferase p300, the coregulator SKIP (Ski-interacting protein), the CDK8-mediator complex, and other mediator complexes, leading to the acetylation of chromatin and upregulation of downstream target genes (reviewed in [55, 56]).

Among various downstream target genes, the major targets of Notch signaling are the hairy/enhancer of split (*Hes*) and the Hes-related (*Hesr/Hey*) family of basic helix-loop-helix (bHLH) transcription factors [57, 58]. These are highly conserved proteins that function as transcriptional repressors. In mammals, well-described Notch target genes include the transcription factors *Hes1*, *Hes5,* and *Hey1* [59]. *Hes1* knockout mice are not viable and have multiple developmental defects [57]. *Hes1* and *Hes5* overexpression in bone marrow partly inhibits B-cell development [60]. *Il2ra* (CD25, IL2-R alpha chain) and *Ptcra* (pre-T-cell receptor alpha chain) are Notch target genes in T cells [61, 62]. Transcription factor *Gata3* is also a direct Notch target gene as a master regulator for T-cell development and later for the Th1/2 lineage decision [63]. *Nrarp* (Notch-regulated ankyrin repeat protein) and *Deltex-1* are two Notch target genes shown as potent negative regulators of Notch signaling [64, 65]. Furthermore, *Myc* (c-myc), *Ccnd1* (cyclin D1), and *Cdkn1a* (p21/Waf1) are Notch target genes implicated in cancer. Other Notch target genes include *Nfkb2*, *Ifi202a*, *Ifi204*, *Ifi205* (*D3*), *Adam19*, *Notch1*, *Notch3*, *Bcl2*, *Tcf3* (E2A), and *Hoxa5*, *9,* and *10* (reviewed in [53]).

## 4. *Drosophila* Oogenesis

*Drosophila* oogenesis as a model system has been used to investigate many aspects of developmental and cell biology. The development of a mature egg from a single stem cell requires almost every cellular process: from cell fate specification, cell cycle control, and cell polarization to epithelial morphogenesis. The *Drosophila* ovary is made up of about 16 to 20 ovarioles, each of which represents an egg production line. Each oocyte develops within a group of cells called an egg chamber (or follicle), which consists of a cluster (or cyst) of 16 germ cells surrounded by somatic follicle cells [66]. At the anterior end of the ovariole is the germarium, which contains somatic and germline stem cells. The germarium is divided into four regions based on morphological differences: regions 1, 2a, 2b, and 3. Egg chambers leave the germarium and mature as they move posteriorly in the ovariole. An ovariole usually contains six to seven sequentially more mature follicles, separated by interfollicular stalk cells. Oogenesis has been divided into 14 stages based on morphological criteria. Stage one is forming of the egg chamber from the germarium, and stage 14 is an egg chamber with a mature egg [67].

The germarium is where new egg chambers are generated. The two germ-line stem cells (GSCs) are located at the anterior end, close to their niche composed of cap cells and terminal filament [68]. The niche prevents GSC differentiation and promotes their self-renewal. The two GSCs alternate in producing one cystoblast at a time. They divide asymmetrically to produce another stem cell and a daughter cell, which begins to differentiate. After four mitotic divisions with incomplete cytokinesis, the daughter cells form a cyst of 16 cells interconnected by cytoplasmic bridges known as ring canals. Each cyst contains eight cells with one ring canal, four with two, two with three, and two with four ring canals. One of the initial two cells with four ring canals, which are called pro-oocytes, will become the oocyte, while all the others become nurse cells. The nurse cells provide nutrients and cytoplasmic components to the oocyte through the ring canals. Within the cyst the oocyte is the only cell that progresses to meiosis. Before exiting the germarium the oocyte arrests in meiotic prophase I, and meiosis does not continue until the mature egg is laid and activated.

The process of oocyte determination occurs gradually as the cyst proceeds through the germarium (reviewed in [69]). Germ cells have a cytoplasmic structure called the spectrosome, which is spherical and contains components of the submembrane cytoskeleton. At the first mitotic division, the spectrosome goes to only one of the two daughter cells. During the following divisions, a branched structure called the fusome is formed when the spectrosome grows from this cell into the other cells [69]. When the cystoblast divides, one pole of the spindle is anchored by the inherited spectrosome (the original fusome), while a new fusome plug forms in the ring canal, at the opposite pole of the cell. The two fusomes then come together to fuse, so that one cell contains the original fusome plus half of the new one, whereas the other cell only retains the other half of the fusome plug. This asymmetric patterning of the fusome is then repeated until the cells finish the fourth division. Therefore the original fusome and more fusome plugs are retained in the oldest cell, in which the fusome always marks the anterior of the cell. This movement of the fusome minimizes the distance between the ring canals. Later on the formation of adherens junctions around the ring canals will stabilize the shape. Most of the fusomes will degenerate by the time oocyte-specific proteins such as BicD (Bicaudal D) or Orb (oo18 RNA-binding protein) accumulated in a single cell in late region 2a. There is a preferential accumulation of the centrosomes as well as of *osk* (oskar) and *orb* mRNAs in this cell with the most fusomes, although it does not rule out the possibility that the other pro-oocyte can become the oocyte too.

As the cysts move through the posterior region of the germarium, they interact with follicle cells. Cysts in the anterior portion of this part of the germarium, known as region 2a, have not been fully enclosed by the follicle cells and still directly contact neighboring cysts [67]. When the cyst gets to region 2b, it changes to a one cell-layer disc and spans the whole width of the germarium, with oocyte-specific factors concentrated in the oocyte and a detectable microtubule-organizing center, which forms a polarized microtubule network that is polarized toward the oocyte and extends into all 16 cells through the ring canals. The somatic or follicle stem cells locate at the junction of regions 2a and 2b and give rise to precursor follicle cells. Sixteen of the precursor follicle cells invade between the cysts, cease division, and develop into polar cells and stalk cells, which play a critical role in follicle formation. The rest of the precursor cells form an epithelial layer around the cyst, producing an egg chamber. The newly formed four to six stalk cells separate the egg chamber from the germarium.

By the time the somatic follicle cells surround the cyst, cell fate markers and markers of meiotic chromosome pairing are restricted to the oocyte, which localizes at the posterior of the egg chambers. As the cyst moves down to region 3 in the germarium (stage 1), a structure called the Balbiani body, which consists of the fusome remnant, mitochondria, centrosomes, a Golgi vesicle, proteins, and mRNAs, is formed at the anterior of the oocyte. At the same time, the cyst rounds up with the oocyte always lying on the posterior edge. As all of the components of the Balbiani body disassociate and move to the posterior cortex, the polarization of the oocyte is established.

When a newly formed egg chamber buds from the germarium, it enters the larger, more posterior region of the ovariole, the vitellarium, consisting of six to seven progressively older follicles. A series of cell-cell signaling events between the germline and the soma and between different populations of somatic cells control the formation of a discrete, correctly polarized and patterned egg chamber. Many signaling pathways play important roles during the course of egg chamber development. Correctly defined and maintained polarity in the oocyte is critical, since this will determine the body axis of the embryo. At the same time, the correctly patterned somatic follicle cells form an intact eggshell and other extraembryonic structures, such as the dorsal appendages.

The establishment of the final anterior-posterior polarity in the oocyte is a two-step process. First, in the germarium *gurken* mRNA, which is part of the Balbiani body, localizes at the posterior of the oocyte as the oocyte polarizes and locates in the posterior end in region 3 [69]. Gurken protein signals to the adjacent follicle cells and results in the adjacent terminal follicle cells developing to a posterior rather than an anterior fate. These posterior follicle cells then induce a repolarisation of the microtubule cytoskeleton in the oocyte at stage 7, which transports the *bicoid* mRNA to the anterior of the oocyte and oskar mRNA to the posterior. By stage 9, the microtubule plus ends accumulate in a compartment at the posterior cortex of the oocyte, with the minus ends predominantly at the anterior region of the oocyte and some extending along the lateral cortex. This microtubule polarity within the oocyte will direct the localization of the RNAs and associated proteins, which define the anterior-posterior axis.

Follicle cells proliferate during stages 1–6 of oogenesis. The egg chamber enlarges during stages 7–9 of oogenesis. The oocyte in the follicle grows significantly, uptakes yolk protein synthesized in the follicle cells, and occupies almost half the egg chamber by stage 10A. Follicle cells cease their mitosis after stage 6 and stay as a cuboidal epithelium through stage 8. The first step of follicular epithelium differentiation is the specification of the terminal follicular cells versus the main body follicular cells. The terminal cells at the anterior pole give rise to the border cells, the stretched cells, and the centripetal cells [70]. These three populations cannot be recognized before stage 9 or 10, when specific genes and proteins are expressed and several morphogenetic features become obvious. In stage 9, reorganization begins through a series of migrations. The majority of the follicle cells stay as a columnar epithelium over the oocyte, leaving around 50 follicle cells as a squamous epithelium over the nurse cells. The 6 to 10 anterior-tip follicle cells become the border cells. They delaminate from the epithelial follicle cells, extend protrusions in between the nurse cells, migrate approximately 100 *μ*m to the border between the nurse cells and the oocyte, and cover the anterior end of the oocyte. During stages 10B to 14, nurse cells contribute maternal mRNAs and proteins to the oocyte by a cytoskeleton-based mechanism and transfer their cytoplasm into the oocyte to help it reach its large size. The follicle cells synthesize the vitelline membrane, then the eggshell over the oocyte. After the completion of the eggshell and the dumping of the nurse cell cytoplasm, the follicle cells and nurse cells undergo apoptosis, leaving behind the mature egg. Specialized follicle cells also make the micropyle for sperm entry. The anterior end of a mature egg also has a pair of dorsal appendages for embryonic respiration and an operculum for larval exit [71].

## 5. Notch Signaling during *Drosophila* Oogenesis

Work by many investigators has shown that Delta-Notch signaling is required for numerous important aspects of oogenesis in *Drosophila melanogaster*. Many of these functions have been studied genetically using mutant alleles of *Notch*, *Delta,* and *Serrate* and by ectopic expression of *Delta* or constitutively activated forms of the Notch receptor.

### 5.1. Germline Stem Cell Niche Formation

 GSC niche formation and maintenance require Notch signaling. Delta and Serrate on the surface of GSCs activate Notch in the somatic cells to form and maintain the GSC niche, and the niche induces and maintains stem cell fate in return [72]. Ectopic or expanded activation of Notch signaling leads to the formation of more cap cells and larger niches, which in turn induce ectopic or more GSCs; conversely, decreased Notch signaling during niche formation results in reduced cap cell number and niche size, and consequently fewer GSCs [73].

### 5.2. Specifying Polar Cells and Stalk Cells

Notch signaling regulates multiple aspects of the differentiation of somatic follicle cells in the *Drosophila* ovary (reviewed in [74]), including differentiation of the stalk and polar cells [75]. This function also involves *fringe* (*fng*), a Notch pathway modulator, which is expressed in the polar/stalk precursors and makes them competent to react to the Delta signal [76]. Loss of *Notch* in follicle cells or of *Delta* in the germ line results in huge fused egg chambers without polar cells. Loss of *fng* also results in egg chambers without polar cells [77, 78]. Loss of *Delta* in the follicle cells results in encapsulation of the cysts by the follicle cells, but stalk formation does not occur. Expression of constitutively active Notch results in more polar cells and long stalks between the egg chambers. The formation of polar and stalk cell fates depends on different levels of Notch activation. The future polar cells have high-level Notch activation, resulting from a germline Delta signal. Stalk cells show low-level Notch activation, which comes from a Delta signal in the polar cells. In polar cells, the metalloprotease Kuzbanian-like (Kul) cleaves and inactivates Delta, reducing the level of Delta signaling so that the stalk precursors next to them can be induced into stalk cells [79]. A recent study has shown that Delta-Notch signaling is required for lateral migration of follicle stem cell daughters across the ovariole as well as for follicle stem cell replacement [80].

### 5.3. Establishment of Anterior-Posterior Polarity

 The formation of the polar and stalk cells occurs by a relay mechanism, which also helps to establish the anterior-posterior axis of *Drosophila* [81]. When a germline cyst reaches region 3 of the germarium, its oocyte has already been positioned to the posterior. Polar/stalk precursors separate the cyst from the adjacent younger cyst in region 2b. Delta signals from the germline cyst activate Notch in the adjacent anterior polar/stalk precursors, inducing them to form polar cells [82]. The more anterior polar/stalk precursors differentiate as stalk cells after receiving a Delta signal from these anterior polar cells. The stalk cells interact with each other and come together toward the middle to form a two cell-wide stalk. This movement forces the younger anterior cyst to round up, being pulled into region 3. At the same time, the stalk induces increased expression of DE-cadherin in the follicle cells that contact the oocyte in the younger cyst, resulting in a preferential adherence between these cells and the oocyte and a posterior position of the oocyte. When the younger cyst finishes all these events, it is in region 3 of the germarium and Delta signaling is activated, which then induces polar cell fate in the polar/stalk precursors in its anterior, and the cycle starts again. During stages 5 to 7, Delta signaling from the germ cells is required for the establishment of anterior-posterior polarity and differentiation of the epithelial follicle cells. Notch mutant epithelial follicle cells at these stages fail to express differentiation markers, resulting in the follicle cells being unable to respond to Gurken by turning on posterior differentiation markers. So without the modulation of Notch signaling, the posterior follicle cells cannot be formed and the anterior-posterior axis of the oocyte is not established [76, 79].

### 5.4. Mitotic Cycle to Endocycle Switch and Differentiation of Epithelial Follicle Cells

 At the end of stage 6, epithelial follicle cells switch from a mitotic cell cycle to a modified cell cycle, called the endocycle, where DNA is duplicated without cell division (endoreplication). Delta in the germline and Notch in the follicle cells are required for this switch [82, 83]. After switching to endoreplicative cycles, follicle cells differentiate by responding to subsequent inductive signals. Notch is required in all epithelial follicle cells for this switch from immature to differentiated follicle cells. Increased Delta expression in the germline at stage 6 is responsible for activation of Notch in the surrounding follicle cells, causing them to switch from mitosis to endoreplication cycles. Follicles mutant for *Notch* at stage 10 display defects in the differentiation of the border, stretched, and centripetal cells, and abnormal migration. At stage 9, Fng-dependent Notch activity is required in the stretched cells and in the most anterior main body follicle cells. Stretched cells require Notch activation to disassemble their adherens junctions for flattening of the stretched cells. Inactivation of Notch signaling in anterior follicle cells, by lack of Fng either alone, or both Dl and Ser, results in clusters of main body follicle cells remaining over the nurse cells [70, 82, 84]. The transcriptional cofactor corepressor for element-1-silencing transcription factor (CoREST) is a newly discovered positive modulator of Notch signaling in somatic follicle cells [85]. Loss of CoREST function in follicle cells disrupted the mitotic-to-endocycle switch at stage 6 of oogenesis. CoREST positively regulates Notch signaling, acting downstream of the proteolytic cleavage of Notch. Subsequent to the mitotic to endocycle switch, main body follicle cells begin synchronized amplification of the chorion genes, which has been termed the endocycle to gene amplification switch. A recent study demonstrates that downregulation of Notch signaling activity plus activation of the ecdysone receptor, acting through the zinc finger protein Tramtrack, is required for the endocycle to gene amplification switch [86].

### 5.5. Migration of Border Cells

The Notch pathway is required for normal border cell migration and is activated in border cells during their migration. Unlike the widespread activation of Notch in follicle cells at stage 6, Notch is only activated in the border cells at stage 9. Expression of Kuzbanian (KUZ), a metalloproteinase that can activate Notch and cleave other substrates, is highly expressed in border cells at the same time [87]. Conditional knockout and/or dominant-negative alleles of KUZ, Notch, and Delta all demonstrate abnormal border cell migration. A dominant-negative form of Kuz decreases Notch activity and inhibits border cell migration without affecting expression of markers of border cell fate or follicle cell differentiation. The ability of the cells to detach from the follicular epithelium is significantly reduced without affecting direction sensing [87, 88].

### 5.6. Centripetal Migration

 By stage 10B high levels of Notch protein accumulate in the centripetal migrating cells, which close off the anterior end of the oocyte while synthesizing the operculum and micropyle. Centripetal migration is blocked in a *Notch* mutant [84]. The expression of the *bunched* (*bun*) gene in the anterior centripetal follicle cells is repressed. In nearby cells, *bun* antagonizes Notch signaling to prevent the posterior cells from differentiating into centripetal follicle cell fates, including gene expression, cell shape changes, and accumulation of cytoskeletal components. *bun* represses *Serrate* and *Delta* expression in posterior follicle cells, coinciding with a boundary of Notch activation in the centripetal follicle cells. Another gene, *slow border cells* (*slbo*), is expressed in centripetally migrating cells as well. At stage 10A, *slbo* expression overlaps *bun* expression in anterior follicle cells; by stage 10B they repress each other's expression to establish a sharp *slbo/bun* expression boundary between migrating and nonmigrating cells. As centripetal migration proceeds from stages 10B to 14, *slbo* represses its own expression and both *slbo* loss-of-function and overexpression mutations reveal that *slbo* is required for centripetal migration. Interactions among *Notch*, *slbo,* and *bun* regulate centripetal migration. The precise position of the *slbo/bun* expression boundary is sensitive to Notch signaling, which is required for both *slbo* activation and *bun* repression [71, 89]. Increased Notch signaling leads to increased *slbo* expression both in the centripetal follicle cells and in adjacent columnar follicle cells. Dynamic interactions among Bun, Slbo, and Notch signaling tightly regulate DE-cadherin levels in the centripetal follicle cells. Absence of DE-cadherin in the germline results in migration of follicle cells between the nurse cells. Migration also is disrupted when follicle cells without DE-cadherin expression are in or near the centripetally migrating follicle cells [71, 84, 89, 90].

### 5.7. Dorsal Appendage Formation

During stages 9–10, dorsal appendage-forming cells are specified by a combination of the BMP, EGF, and Notch pathways [71, 91]. By stage 11, these epithelial cells can be found at the dorsoanterior corner of the egg chambers. They constrict apically and move inside-out, turning from a flat layer into tubular structures. The appendages are formed after the secretion of chorionic proteins into the tube lumens. Notch signaling plays an important role in establishing a boundary between the *Rhomboid*- and the *Broad*-positive cells, which form the dorsal and ventral portions of the dorsal appendage tube. A difference in Notch levels in adjacent cells is critical for this process. At the boundary, cells with high Notch levels express *Rhomboid*, whereas cells with lower Notch express *Broad*. When Notch is absent in cells that span the boundary, *Rhomboid* is not expressed, and *Broad* is ectopically expressed. Therefore Notch signaling regulates the patterning of both *Rhomboid*- and *Broad*-positive cell types at the boundary. The establishment of this border is important for preventing intermingling of these cell types during tube formation [71, 92]. In a similar manner to their cooperation during the endocycle to gene amplification switch [86], the Tramtrack transcription factor, Notch signaling, and ecdysone receptor activation cooperate to control the volume of the dorsal appendage tubes by promoting apical reexpansion and lateral shortening of dorsal appendage-forming follicle cells [93].

## 6. Perspectives

The studies described in the preceding section clearly demonstrate the multiple important roles played by the Notch signaling pathway during *Drosophila* oogenesis. An unanswered question, however, is whether a critical role for Notch signaling during oogenesis exists in other organisms, such as mammals. *In situ* hybridization analyses of adult mouse ovaries demonstrated that the *Notch2*, *Notch3,* and *Jag2* genes are expressed in granulosa cells (the somatic cells of the ovarian follicle), and the *Jag1* gene is expressed in the oocytes [94]. Furthermore, the Notch target genes *Hey1* and *Hey2* are also expressed in the somatic follicle cells. A similar pattern of expression was observed during the early neonatal period, when ovarian primordial follicles are assembled [95]. Culture of neonatal mouse ovaries in *γ*-secretase inhibitors led to defects in the early stages of follicle development [95, 96]. These data indicate that Notch signal reception is occurring in the somatic follicle cells of the mouse ovary and are consistent with the model that Notch signal reception in granulosa cells is essential for ovarian follicle development. However, *γ*-secretase inhibitors have many substrates in addition to Notch family receptors [97]. Genetic analyses, such as oocyte- and granulosa cell-specific deletion of Notch ligands and receptors in mutant mice, will be required to confirm a role for Notch signaling during mammalian oogenesis and to determine which Notch pathway components are essential for this process.
